# The enigmatic SAR202 cluster up close: shedding light on a globally distributed dark ocean lineage involved in sulfur cycling

**DOI:** 10.1038/s41396-017-0009-5

**Published:** 2017-12-05

**Authors:** Maliheh Mehrshad, Francisco Rodriguez-Valera, Mohammad Ali Amoozegar, Purificación López-García, Rohit Ghai

**Affiliations:** 1 Institute of Hydrobiology, Department of Aquatic Microbial Ecology, Biology Centre of the Academy of Sciences of the Czech Republic, České Budějovice, Czech Republic; 2 Evolutionary Genomics Group, Universidad Miguel Hernández, San Juan de Alicante, Spain; 3 Extremophiles Laboratory, Department of Microbiology, Faculty of Biology and Center of Excellence in Phylogeny of Living Organisms, College of Science, University of Tehran, Tehran, Iran; 4 Ecologie, Systématique, Evolution, CNRS, Université Paris-Sud, Université Paris-Saclay, AgroParisTech, Orsay, France

## Abstract

The dark ocean microbiota represents the unknown majority in the global ocean waters. The SAR202 cluster belonging to the phylum *Chloroflexi* was the first microbial lineage discovered to specifically inhabit the aphotic realm, where they are abundant and globally distributed. The absence of SAR202 cultured representatives is a significant bottleneck towards understanding their metabolic capacities and role in the marine environment. In this work, we use a combination of metagenome-assembled genomes from deep-sea datasets and publicly available single-cell genomes to construct a genomic perspective of SAR202 phylogeny, metabolism and biogeography. Our results suggest that SAR202 cluster members are medium sized, free-living cells with a heterotrophic lifestyle, broadly divided into two distinct clades. We present the first evidence of vertical stratification of these microbes along the meso- and bathypelagic ocean layers. Remarkably, two distinct species of SAR202 cluster are highly abundant in nearly all deep bathypelagic metagenomic datasets available so far. SAR202 members metabolize multiple organosulfur compounds, many appear to be sulfite-oxidizers and are predicted to play a major role in sulfur turnover in the dark water column. This concomitantly suggests an unsuspected availability of these nutrient sources to allow for the high abundance of these microbes in the deep sea.

## Introduction

The marine habitat is the largest on Earth and marine microbes play fundamental roles in global biogeochemical nutrient cycling [1]. Planktonic communities are permanently stratified by light into two different zones with remarkably different microbial compositions. The photic zone occupies the uppermost ca. 200 m and is characterized by intense photosynthetic activity amidst steep gradients of light intensity, nutrients and temperature. The far larger aphotic zone extends all the way down to the sea floor and is permanently dark, cold, oligotrophic and heavily dependent upon organic input from the more productive layers above. Microbial communities are further stratified within these two zones. For instance, a clear stratification is observed among several planktonic cyanobacterial genera, *Prochlorococcus* being located below the *Synechococcus* maxima in the photic zone [2, 3]. When spatio-temporal distributions of microbes in the water column of the marine habitat began to be explored by 16S rRNA sequences, novel and widespread microbial groups were discovered. e.g., SAR11 [4] and mesophilic group I (now *Thaumarchaeota*) and II archaea [5, 6]. Among the early discoveries was a group of 16S rRNA sequences named SAR202 cluster that affiliated to the phylum *Chloroflexi* and had no previously described representatives in the marine habitat [7]. These sequences were found preferentially at the lowermost bounds of the photic zone (depth 250 m) in the North Atlantic and Pacific oceans. Additional SAR202 sequences were retrieved in 16S rRNA clone surveys in deep waters of the Pacific Ocean (3000 m), extending the presence of this group to the deep- sea [8]. Similar sequences were also retrieved from the deep, cold hypolimnion (500 m) of an oligotrophic lake (Crater Lake) [9]. Unequivocal evidence for the widespread prevalence of the SAR202 throughout the mesopelagic and bathypelagic realms was provided by fluorescence in-situ hybridization (FISH), revealing high numbers of SAR202 cells throughout the aphotic water column ( > 10% of total prokaryotic community), down to nearly 3500–4000 m in the Atlantic and Pacific oceans [10, 11].

Despite the SAR202 group being among the first widespread microbial lineages discovered in the aphotic zones [7] we still lack cultured representatives for this group and their physiological characteristics remain completely unknown. In-situ substrate uptake experiments suggested that a large fraction of SAR202 cells utilize L-aspartic acid at all depths in favor of the more refractory D-asp. This is in contrast to the global bacterial and archaeal community that shows decreased uptake rates for L-Asp with increasing depth, suggesting adaptability of SAR202 to available dissolved organic matter in the deep sea [12]. A metagenomic analysis of the deep Mediterranean Sea water column also reported a high abundance of *Chloroflexi* in the deep sea and suggested the potential for carbon-monoxide oxidation in these microbes [13, 14]. FISH analyzes showed that SAR202 cells have a coccoid morphology with a diameter of > 1 µm [10] that does not vary with depth. This is unlike SAR11, whose cell-size generally increases with depth [11], something that correlates with a slight increase in genome size for deep SAR11 ecotypes [15]. SAR202 does not show strong correlation to environmental parameters (except perhaps to lower temperatures and increasing depth). Reports are sometimes conflicting in this respect, e.g., correlation to high oxygen concentrations found by Giovannoni et al. [7] and lack thereof by Schattenhofer et al. [11].

This in itself is quite remarkable for a group that at some sites may comprise > 40% of the total bacterial community in the deep oceans [12]. Phylogenetic analysis of 16S rRNA genes in general provides little information regarding metabolic traits. This is particularly true for *Chloroflexi*, which display a broad spectrum of lifestyle characteristics, e.g., anoxygenic photosynthesizers [16, 17], obligate aerobic/anaerobic heterotrophs [18–21], thermophiles [18, 20, 22], halophiles [23], nitrite oxidizers [24, 25], predators with gliding motility [26], some with capacity for reductive dehalogenation [27, 28] and even endospore-forming Gram-positive bacteria in a largely Gram-negative phylum [29, 30]. The only genomic information from the SAR202 group at present is from an incomplete single cell amplified genome (SAGs), Pac-SCGC-AAA240-N13, obtained from a depth of 770 m from the ALOHA station in the Pacific Ocean north of Hawaii [31] but no genomic analyzes have been reported.

In this work, we reconstructed several SAR202 genomes from deep marine and brackish metagenomics datasets from the Ionian, Aegean and the Caspian Sea as well as Pacific, Atlantic and Indian oceans. By screening publicly available unclassified *Chloroflexi* genomes we also identified another SAG, Atl-SCGC-AB-629-P13, (obtained from a depth of 553 m in the mid-Atlantic) as belonging to the SAR202 lineage. These metagenome-assembled SAR202 genomes (MAGs), together with the two SAGs provide a first insight into the metabolic capabilities of this group and suggest a significant role in the deep-ocean sulfur cycle. Moreover, metagenomic fragment recruitment analyzes reveal that highly related SAR202 genomes are prevalent at geographically distant locations (from the Mediterranean to the Pacific) implying their global distribution as a deep stratified cluster with a sizeable contribution to global ocean biogeochemical cycles, particularly the sulfur cycle.

## Material and methods

### Metagenomic datasets used in this study

The metagenomic samples from the Caspian Sea were taken at the peak of stratification in October 2013 at three depths (15 m, 40 m and 150 m, bottom depth 230 m). Sample collection, DNA extraction, sequencing and assembly of these datasets are described in more detail in Mehrshad et al. Mehrshad et al. [32] and the sequence data are available from NCBI SRA (Bioproject PRJNA279271). The two deep sea samples from the Mediterranean were collected in the Aegean Sea (600 m, bottom depth 699 m) and Ionian Sea (3500 m, bottom depth 3633 m) in October 2010. These datasets and additional deep chlorophyll maximum samples used in this study are available in NCBI SRA (Bioprojects PRJNA305355 and PRJNA257723). Three representative deep, marine metagenomes from the MALASPINA expedition were also used for assembly (SRR3965592, SRR3963457, and SRR3961935). The Caspian and the Mediterranean metagenomes from all samples analyzed in this study are from the 0.22–5.0 µm fraction and were sequenced by HiSeq2000 (paired end reads of length 100 bp). The MALASPINA datasets are from the 0.2 to 0.8 µm fraction and were sequenced using HiSeq2000 (paired end reads of length 150 bp). Basic metadata (sampling date, latitude, longitude, depth, bioproject identifiers, SRA accessions), sequence statistics (number of reads, read length, dataset size) and % of *Chloroflexi* assigned 16S rRNA reads identified in all metagenomes used for assembly are provided in Supplementary Table S1.

### Unassembled 16S rRNA read classification

A non-redundant version of the RDP database [33] was created by clustering its ca. 2.3 million 16S rRNA gene sequences into approximately 800,000 sequences at 90% nucleotide identity level using UCLUST [34]. A 20 million subset of the reads from the Illumina datasets was compared to this reduced set and an e-value cutoff of 1e-5 was used to identify candidate 16S rRNA gene sequences. The candidate sequences were further examined using ssu-align, to separate them into archaeal, bacterial, and eukaryotic 16S/18S rRNA or non-16S rRNA gene sequences [35]. Only bona fide 16S rRNA sequences were finally compared to the complete RDP database and classified into a high level taxon if the sequence identity was ≥ 80% and the alignment length was ≥ 90 bp. Sequences failing these thresholds were discarded. To get an overview of the distribution of 16S rRNA reads assigned to Chloroflexi across the global ocean, in addition to the Mediterranean (DCM and Deep), Caspian [32] and MALASPINA [36] datasets, we also used surface, DCM and mesopelagic datasets generated by the TARA expedition [37].

### Metagenome assembly and annotation

All three datasets retrieved from depth profile of the Caspian Sea (15 m, 40 m and 150 m) were assembled together (using IDBA_UD) [38] as described previously [32]. The Mediterranean DCM datasets, the Deep Mediterranean and the MALASPINA datasets were quality trimmed using sickle (https://github.com/najoshi/sickle, default parameters) and assembled using the megahit assembler (--k-min 39 --k-max 99 --k-step 10 --min-count 2) [39]. The MALASPINA datasets were chosen because they had a high percentage of *Chloroflexi* 16S rRNA reads (9–11%) (Supplementary Table S1), and also because they represented geographically distant locations (South Atlantic, Indian Ocean and North Pacific).

Prodigal (in metagenomic mode) was used for predicting protein coding genes in the assembled contigs [40]. tRNA prediction was performed using tRNAscan-SE [41] and ribosomal rRNA genes were identified with meta_rna [42]. Functional gene annotation was performed by comparisons against COG hmms [43] using and e-value cutoff of 1e-5, and the TIGRfams models [44] (using trusted score cutoffs --cut_tc) using the hmmer package [45]. The assembled genomes were also annotated using the RAST server [46] and BlastKOALA [47]. Additionally, all predicted proteins were also compared to the NCBI-NR database using BLASTP at e-value 1e-5.

### Genome reconstruction

Only contigs longer than 10 kb were used in the genome reconstructions. A contig was considered to belong to the phylum Chloroflexi if a majority of its genes gave best BLAST hits to this phylum. Contigs within each dataset were grouped using taxonomic affiliation, principal component analysis of tetranucleotide frequencies, GC% and coverage in different metagenomes as described previously [48–50]. Tetranucleotide frequencies were computed using the compseq program (-word 4 -reverse) in the EMBOSS package (Rice et al. 2000). Principal components analysis was performed using the FactoMineR package (function res.pca()) in R [104]. Enzyme EC numbers were predicted using PRIAM [51] and metabolic pathway reconstruction was performed using Pathway Tools [52].

### Metagenomic fragment recruitmen**t**

To avoid bias in recruitment results owing to the presence of highly related rRNA sequences, rRNA sequences in genomes were masked. After masking, recruitments were performed using BLASTN [53], and a hit was considered only when it was at least 50 nucleotides long, had a nucleotide identity of > 95% and an e-value of ≤ 1e-5. These cutoffs approximate species-level divergence [54]. These hits were used to compute the RPKG (reads recruited per kilobase of genome per gigabase of metagenome) values that reflect abundance values and are normalized and comparable across different genomes and metagenomes (RPKG = Number of reads matching a contig or genome per Kb/Total number of bases in the metagenome expressed in Gb). In order to confirm that the reads assigned to SAR202 genomes using these cutoffs did not contribute to abundance of other microbes, we also compared the SAR202 assigned reads from three bathypelagic MALASPINA datasets (10 million reads each from SRR3961935, SRR3963457, SRR3965592) to the complete RefSeq collection (ca. 5000 microbial genomes). None of the SAR202 assigned reads matched any genome in RefSeq confirming that these cutoffs are specific for detecting only reads originating from genomes of the SAR202 cluster.

**Screening for SAR202 cluster genomes**. All publicly available *Chloroflexi* genomes from NCBI and JGI were downloaded. Phylogenetic analyzes of 16S rRNA sequence and conserved concatenated proteins suggested that two single cell amplified genomes SCGC-AB-629-P13 (Mid-Altlantic Ocean, 553 m depth) and SCGC-AAA240-N13 (Pacific Ocean, ALOHA station, Hawaii, 770 m depth) also belonged to the SAR202 group and were included in all analyzes (Supplementary Figs. 3 and 5).

### Phylogenomics and genome size estimation

**Fig. 1:**
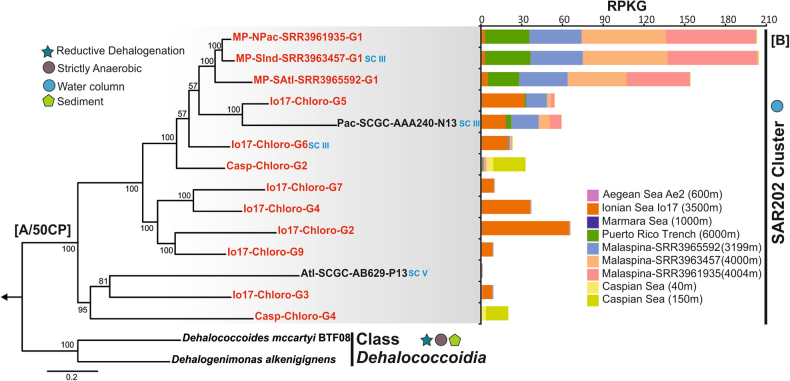
**a** Maximum likelihood phylogeny of the 12 almost complete reconstructed genomes from Caspian, Aegean, Ionian Seas and MALASPINA datasets in the SAR202 cluster together with the single cell amplified genomes of the SAR202 cluster. The tree was made using a concatenate of 50 conserved proteins. Genomes of the class *Dehalococcoidia* were used to root the tree. The reconstructed genomes from this study are highlighted in red. The subcluster designation for genomes containing 16S rRNA is shown in blue (SC III and SC V). Bootstrap values (%) are indicated at the base of each node. Legends for lifestyle hints are on top left. **b** Metagenomic recruitment of almost complete reconstructed genomes together with available single cell genomes of SAR202 cluster in different deep datasets from brackish and marine environments. Brackish datasets include two aphotic depths of the Caspian Sea. Marine datasets include Aegean, Ionian, and Marmara Sea deep datasets, and Puerto Rico Trench deep dataset, together with three of the deep MALASPINA datasets also used for assembly. The depth of samples for each dataset is mentioned inside parenthesis

A reference phylogenomic tree was made using PhyloPhlAN [55], the complete genomes of representatives from all known Chloroflexi classes and reconstructed MAGs of this study were added to the built-in tree of life in PhyloPhlAN (Supplementary Fig. S5). The two single cell amplified genomes SCGC-AB-629-P13 (Mid-Altlantic Ocean, 553 m depth) and SCGC-AAA240-N13 (Pacific Ocean, ALOHA station, Hawaii, 770 m depth) were also added as confirmed SAR202 representatives verified by 16S rRNA analysis (Supplementary Fig. S3). PhyloPhlAN uses USEARCH [34] to identify the conserved proteins and subsequent alignments against the built-in database are performed using MUSCLE [56]. Finally, an approximate maximum-likelihood tree is generated using FastTree [57] with local support values using Shimodaira–Hasegawa test [58]. This analysis confirmed that all MAGs belong to the phylum *Chloroflexi* and are phylogenetically most related to the SAR202 cluster branching together with the two confirmed SAR202 SAGs. For creating the sub-tree (Fig. 1) of MAGs with higher than 50% completeness, only *Dehalococcoidia* genomes were used as outgroups (as they are the closest neighbors of SAR202 (Supplementary Fig. S5) to reveal better the internal phylogenetic relationships within the incomplete genomes by maximizing the number of common proteins. For creating the sub-tree phylogeny shown in Fig. 1, 50 conserved proteins in the reconstructed genomes and the reference genomes were identified using the COG database [43]. These proteins were concatenated and aligned using Kalign (default parameters) [59] and the alignment was trimmed using trimAL (--gappyout) [60]. A maximum-likelihood tree was constructed with FastTree2 [57], using a JTT + CAT model, a gamma approximation, and 100 bootstrap replicates.

Two sets of previously described genes, one with 35 single copy orthologous genes [61], and another with 112 essential genes [48] found in bacteria and also a set of 140 common genes (identified using TIGRFams [62] between 41 complete *Chloroflexi* genomes) were used to estimate genome completeness (Supplementary Table S2). Additionally, CheckM [63] was also used to estimate genome completeness and provided very similar results to the genome size estimation performed using 140 common *Chloroflexi* genes (Supplementary Table S3).

### Single gene phylogeny and ANI

16S rRNA sequences from Chloroflexi genomes, clone libraries, SAGs and MAGs were aligned using MUSCLE [56], and a maximum-likelihood tree was constructed with FastTree2 [57], using a GTR model, a gamma approximation, and 100 bootstrap replicates. The *apr* protein sequence alignments were also performed using MUSCLE [56], and FastTree2 [57], was used for creating the maximum-likelihood tree (JTT + CAT model, gamma approximation, 100 bootstrap replicates). Average Nucleotide Identity (ANI) was calculated as defined in Ref. [54].

### Accession numbers

The assembled genomic bins have been deposited to DDBJ/EMBL/GenBank and can be accessed under the Bioproject PRJNA356693 and the accession numbers MUCH00000000, MUCI00000000, MUCJ00000000, MUCK00000000 (for Casp-Chloro-G1-4), MUCL00000000, MUCM00000000, MUCN00000000 (for Ae2-Chloro-G1-3), MUCO00000000, MUCP00000000, MUCQ00000000, MUCR00000000, MUCS00000000, MUCT00000000, MUCU00000000, MUCV00000000, MUCW00000000 (for Io17-Chloro-G1-9), MUCX00000000, MUCY00000000 (for MP-SAtl-SRR3965592-G1 and –G2), MUCZ00000000, MUDA00000000 (for MP-SInd-SRR3963457-G1 and –G2), MUDB00000000 (for MP-NPac-SRR3961935-G1).

## Results and discussion

### SAR202 abundance in marine and brackish environments

A broad overview of presence of phylum *Chloroflexi* representatives in the water column of marine and brackish environments using 16S rRNA metagenomic reads is shown in Supplementary Fig. S1A (See also Supplementary Table S4). While SAR202 were barely detectable in the photic zone and deep chlorophyll maximum (DCM) datasets (Western and Eastern Mediterranean, Caspian and Red Sea, HOTs and BATs), they comprised a substantial fraction of the microbial community in the datasets from the deep aphotic zones of eastern Mediterranean (Aegean ca. 5% and Ionian ca. 10%). The Caspian datasets also showed a progressive increase in the percentage of reads assigned to *Chloroflexi* with increasing depth, reaching a maximum of ca. 4% at 150 m, suggesting the existence of novel brackish SAR202 representatives. In comparison, the Red Sea depth profile did not show any significant amounts of *Chloroflexi* members (maximum sample depth 500 m), suggesting that SAR202 does not show an ecological preference for the particular physico-chemical conditions characterizing the dark Red Sea [64, 65]. An integrated view of the distribution of 16S rRNA reads assigned to Chloroflexi in metagenomic datasets from the surface, DCM, mesopelagic and bathypelagic ocean layers shows clearly an increasing abundance with depth (Supplementary Fig. S1B). There are statistically significant differences between the surface-mesopelagic, surface-bathypelagic datasets, DCM- mesopelagic and DCM-bathypelagic datasets (Kruskal–Wallis test, *P* = <0.001). Apart from the deep Mediterranean, the high abundance of *Chloroflexi* across different geographical locations in the global ocean and depths in the Pacific, Atlantic and Indian Oceans reaffirms the deep dark waters of global oceans as their preferred niche (Supplementary Fig. S1 and Supplementary Table S3). *Chloroflexi* even comprised up to 14% of microbial community in some deep ocean datasets of the MALASPINA expedition. They appear to be present in variable proportions at all deep aphotic zones for which data are available (largely −40 N to 44 S) without obvious preferences. Only one deep ocean dataset is available from the Antarctic Province that is outside these latitudinal bounds (TARA expedition, 790 m depth, −61.98 S, −49.36 W) and it also contained 3.35 percent of 16S rRNA reads of *Chloroflexi* (Supplementary Fig. S2). Overall, these analyzes recapitulate previous findings indicating that the SAR202 abundance increases with depth [7, 10–12, 14] and has a global distribution in the marine water column.

### Genome reconstruction and phylogenomics

In order to obtain a first insight into the genomic repertoire and diversity of the SAR202 group, we chose a number of marine and brackish metagenomic datasets for assembly, annotation and taxonomic binning (Supplementary Table S1, see also Methods). These include two deep datasets from the Eastern Mediterranean (Aegean and Ionian Sea) and three representative deep datasets from the MALASPINA expedition (one each from South Atlantic Ocean, Indian Ocean and North Pacific Ocean). To identify brackish SAR202 representatives, existing assemblies from the Caspian Sea were used [32]. We also included for assembly five deep chlorophyll maximum datasets from the Mediterranean. After the assembly, we selected long contigs ( > 10 Kb) in which the majority of predicted proteins gave best hits to *Chloroflexi* genomes. As expected from the 16S rRNA results, very few *Chloroflexi* contigs could be identified in the DCM datasets as *Chloroflexi* are only minor constituents of the photic zone communities (Supplementary Fig. S1 and Supplementary Table S5) and they were not considered further. The Ionian Sea (Io17) datasets from 3500 m depth had the highest number of assembled *Chloroflexi* affiliated genomic fragments (709 contigs) with total size of 17.8 Mb (Supplementary Table S5). Using differences in GC content, coverage in different datasets, and principal components analysis of tetranucleotide frequencies, (see Methods) these contigs were further segregated to 21 genomic bins (nine from the Ionian, three from Aegean, four from the Caspian, two each from South Atlantic and South Indian Ocean and one from the North Pacific Ocean, Supplementary Table S5). Although, like all other metagenomic assemblies, these bins are not “clonal”, we will refer to them as “genomes” or MAGs (metagenome-assembled genomes). We also identified two single-cell amplified genomes (SAGs) that also belong to the SAR202 cluster in a comparison with these metagenome-assembled genomes (Atl-SCGC-AB-629-P13, Mid-Atlantic Ocean, 553 m depth and Pac-SCGC-AAA240-N13, Pacific Ocean, ALOHA Station, Hawaii, 770 m depth). Detailed statistics of these reconstructed *Chloroflexi* genomic bins and the two SAGs are shown in Table 1. Three genomic bins contained contigs harboring partial 16S rRNA genes (Io17-Chloro-G6 – 333 bp, MP-SInd-SRR3963457-G1 – 947 bp, and MP-SAtl-SRR3965592-G2 – 926 bp) and all could be unequivocally affiliated to the SAR202 cluster (subcluster III) within the phylum *Chloroflexi* (Supplementary Fig. S3) to which the original SAR202 clones also belong [10]. A nearly complete 16S rRNA gene was also found in the SAG Pac-SCGC-AAA240-N13 (1508 bp), which also affiliated to subcluster III. Only a single genome (SAG Atl-SCGC-AB-629-P13, 16S rRNA 1202 bp) was found to belong to subcluster V. The average nucleotide identity (ANI) comparison of all the MAGs and SAGs of SAR202 cluster shows that the five MAGs from MALASPINA datasets belong to two different species (Supplementary Fig. S4).

**Table 1:** Summary statistics of assembled *Chloroflexi* genomic bins in SAR202 cluster

	#contigs	GC%	length (Mb)	#CDS	% Completeness	Est. genome size range (Mb)
Metagenomic assembled genomes (MAGs)						
Caspian Sea (15, 40, and 150 m)						
Casp-chloro-G1	20	68	0.353	346	–	–
Casp-chloro-G2	66	61	1.9	1775	69–89	2.1–2.75
Casp-chloro-G3	60	56.1	1.36	1296	64–82	1.65–2.1
Casp-chloro-G4	53	55.5	1.36	1301	61–77	1.8–2.2
Aegean Sea (Ae2, 600 m)						
Ae2-chloro-G1	28	43.9	0.465	462	–	–
Ae2-chloro-G2	45	44.2	0.615	605	–	–
Ae2-chloro-G3	32	54.4	0.476	444	–	–
Ionian Sea (Io17, 3500 m)						
Io17-chloro-G1	40	55.1	0.875	855	46–68	1.3–1.9
Io17-chloro-G2	50	58.7	1.56	1401	64–79	1.97–2.4
Io17-chloro-G3*	100	47.5	2.4	2319	84–93	2.6–2.8
Io17-chloro-G4	69	55.7	1.9	1767	71–88	2.2–2.7
Io17-chloro-G5	44	57	1.55	1449	61–79	1.97–2.5
Io17-chloro-G6	49	58.3	2.2	2076	74–91	2.4–2.9
Io17-chloro-G7	74	54.8	1.45	1277	57–79	1.8–2.5
Io17-chloro-G8	51	58.7	1.05	1005	50–71	1.5–2.1
Io17-chloro-G9*	200	59	4.3	4053	81–95	2.3–2.7
MALASPINA, South Atlantic gyral province (SRR3965592, 3199 m)						
MP-SAtl-SRR3965592-G1	119	59.2	1.9	1944	64 - 85	2.3 – 3.02
MP-SAtl-SRR3965592-G2	39	57.6	0.568	532	–	–
MALASPINA, Indian south subtropical gyre province (SRR3963457, 4000 m)						
MP-SInd-SRR3963457-G1	54	59.3	2.04	1964	71 - 90	2.3 – 2.9
MP-SInd-SRR3963457-G2	52	57.5	0.756	754	–	–
MALASPINA, North Pacific tropical gyre province (SRR3961935, 4004 m)						
MP-NPac-SRR3961935-G1	54	59.1	2.09	1998	67–89	2.3–3.1
Single cell amplified genomes (SAGs)						
Pacific Ocean (ALOHA station, 770 m)						
SCGC-AAA240-N13	215	55	1.45	1565	43–62	2.3–3.4
Atlantic Ocean (mid-Atlantic, 553 m)						
SCGC-AB-629-P13	64	41.4	0.84	862	59–71	1.2–1.4

*An asterisk next to a genome indicates a bin containing more than one copy of the genome

*Gray rows indicate small genomes ( < 50% average completeness)

The MAGs, SAGs and the previously available Chloroflexi genomes were inserted into a reference tree of life (3174 microbial genomes) using PhyloPhlAn [55]. All MAGs and SAGs formed a large “Class” level group with two distinct sub-clusters within the phylum *Chloroflexi* (Supplementary Fig. S5 and Fig. 1). This was possible for all genomes except Caspian-Chloro-G1 because of the small size (only 0.35 Mb) and lack of common conserved proteins although the predicted proteins in this MAG gave best blast hits to SAR202 members. The closest neighbors of the SAR202 group appear to be the class *Dehalococcoidia*, whose cultivated representatives are mostly sediment-dwelling anaerobes (freshwater or marine) [28, 66, 67]. This relatedness has been seen in the 16S rRNA phylogeny as well (Supplementary Fig. S3) [10]. Of the few sequenced genomes that are available from the genus *Dehalococcoidea*, it appears they have streamlined, small genomes with size in the range 1.5–1.8 Mb (genomic GC 45-65%). Several studies showed that they are capable of organohalide respiration (particularly reductive dechlorination) [28, 68–70]. However, recently this ability has been also expanded to some members of the class *Anaerolineae* of the phylum *Chloroflexi* [71]. In what follows, we focused our analyzes largely on the twelve most complete MAGs (seven genomes from deep Ionian Sea, two genomes from the Caspian Sea, one genome each from assembled deep MALASPINA datasets, MP-NPac-SRR3961935, MP-SInd-SRR3963457, and MP-SAtl-SRR3965592) and the two SAR202 cluster SAGs.

### SAR202 global distribution and vertical stratification

All MAGs reconstructed in this study showed highest recruitment values in their environment of origin along with higher abundances in the deeper strata of the water column (Fig. 1 and Supplementary Fig. S6). Similar trends were observed in the global ocean, where the surface and deep chlorophyll maximum datasets were largely devoid of SAR202 representatives, but the deep aphotic datasets showed a far higher abundance for reconstructed MAGs and SAGs of the SAR202 cluster (Supplementary Fig. S7A).

Nine SAR202 genomes, including three from the Ionian (Io17-G1, Io17-G5, Io17-G8, 3500 m), two each from the South Atlantic (MP-SAtl-SRR3965592-G1 and –G2, 3199 m) and Indian Ocean (MP-SInd-SRR3963457-G1 and –G2, 4000 m), one from North Pacific (MP-NPac-SRR3961935-G14004 m depth) and Pac-SCGC-AAA240-N13 (770 m depth, Pacific Ocean) appear to be widely distributed in several deep aphotic datasets (Supplementary Figs. S6 and S7B).

**Fig. 2:**
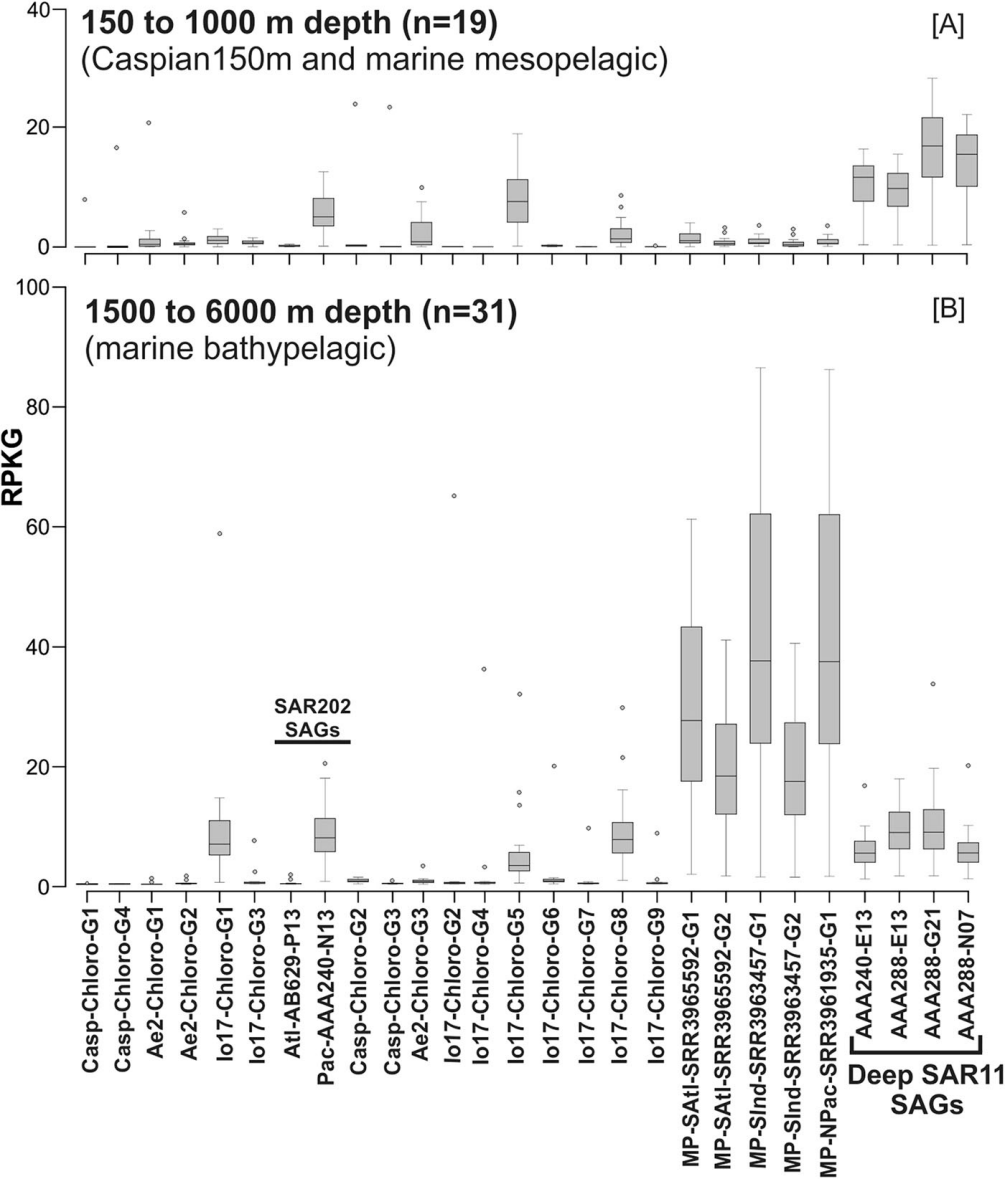
Overview of the recruitment (RPKG) distribution of all genomes in SAR202 cluster together with deep specific single cell amplified genomes of the SAR11 clade. **a** Recruitment RPKG distribution in marine mesopelagic datasets together with the deep Caspian dataset from 150 m depth. Datasets cover the depths from 150 m to 1000 m (*n* = 19) in the mesopelagic layer and they include Caspian Sea (150 m), TARA mesopelagic (550–1000 m), Aegean Sea (600 m) and Marmara Sea (1000 m). **b** Recruitment (RPKG) distribution in marine deep datasets in the range of 1500–6000 m (*n* = 31) including deep MALASPINA datasets together with the deep sea datasets of the Ionian Sea (3500 m) and Puerto Rico deep trench (6000 m). The black dots in the box plots are outlier values and the line inside the boxes indicates the median value

However, on segregating datasets into mesopelagic (up to 1000 m deep, 19 datasets) and bathypelagic (1500–6000 m, 31 datasets) a different recruitment pattern is evident. While Ae2-Chloro-G1, Ae2-Chloro-G3, Io17-Chloro-G5 recruited more reads in the mesopelagic datasets, Io17-Chloro-G1, Io17-Chloro-G8, all MALASPINA genomes and Pac-SCGC-AAA240-N13 recruited even more from the bathypelagic datasets (Fig. 2). This differential recruitment in meso- and bathypelagic layers of the ocean suggests a vertical stratification of SAR202 in the aphotic zone. It has been suggested that the mesopelagic layer is more influenced by the upper layers and the bathypelagic realm has a comparatively more stable status regarding different physicochemical features [72–75]. Despite the distant origin of the genomes from the MALASPINA datasets, they show high recruitment values in almost all available deep bathypelagic datasets suggesting the presence of the same species in widely different geographic areas (Southern Indian Ocean, South Atlantic, North Pacific Ocean). Remarkably, a differential vertical stratification was also seen for currently available deep specific SAR11 SAGs, (isolated from the mesopelagic zone, 770 m depth), that are more abundant in the mesopelagic than in bathypelagic datasets (Fig. 2). These deep specific SAR11 SAGs appear also to be much more abundant in the mesopelagic samples than the cultured isolates in the photic zone (Supplementary Fig. S8).

However, another pattern is also evident for some SAR202 genomes, with high abundances at the place where the original metagenome was obtained from, and lower at others. These observations likely reflect biogeographic patterns resulting from local environmental selection. Nevertheless, these results suggest that at least some SAR202 genomes recovered here are primarily bathypelagic, extremely abundant, much more than the currently available SAR11 representative genomes are (either from deep or surface), being among the most abundant microbial genomes retrieved from the aphotic zone at large. The brackish SAR202 MAGs reconstructed from metagenomes of the Caspian Sea also display a clear vertical stratification similar to the marine habitat along the Caspian depth profile datasets (Supplementary fig. S6) although at shallower depths than their marine bathypelagic counterparts.

### Metabolism

**Fig. 3:**
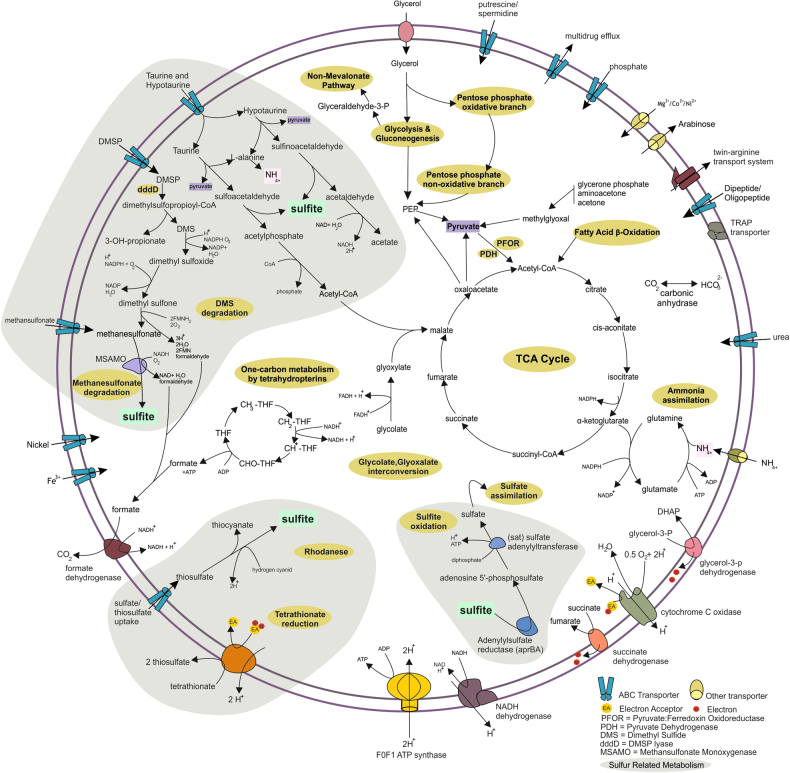
Mosaic schematic view of the metabolic traits in all available genomes from SAR202 cluster. The parts affiliated with sulfur compound metabolism are highlighted in gray

**Fig. 4:**
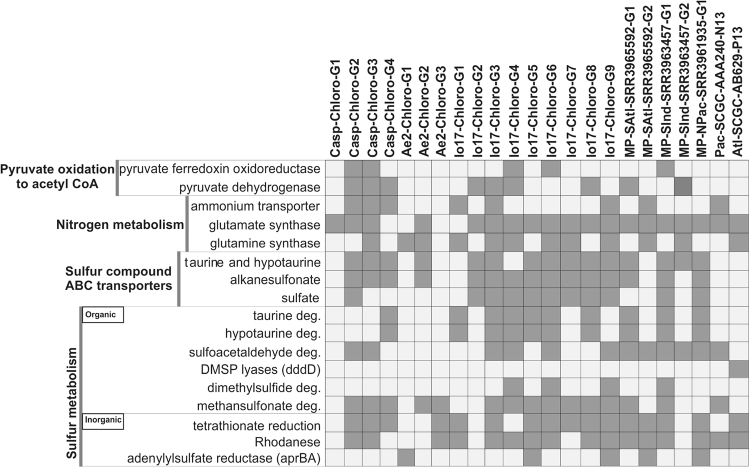
Overview of the metabolic highlights of the MAGs and SAGs of the SAR202 cluster. Filled squares indicate the presence of the pathway/gene

Genome annotation of SAR202 members suggests they are capable of organo and lithotrophic metabolisms. Representative genes for central carbohydrate metabolism like tricarboxylic acid cycle, pentose phosphate, glycolysis and gluconeogenesis pathways were present (see Fig. 3 and Fig. 4). However, both aerobic and anaerobic routes for pyruvate oxidation to acetyl CoA, via pyruvate dehydrogenase (PDH; aerobic) and pyruvate ferredoxin oxidoreductase (PFOR; anaerobic) were found, similar to other Chloroflexi genomes [76] [102]. No evidence for autotrophic carbon fixation was found in any of these genomes.

Almost all MAGs and SAGs of the SAR202 cluster encode genes responsible for ammonia assimilation as a part of their nitrogen metabolism (e.g., ammonium transporter, glutamate synthase, glutamine synthase, all key genes for ammonia assimilation) (See Figs. 3 and 4). In addition, SAR202 seem to utilize other potential nitrogen sources in the deep sea such as hypotaurine and taurine, that function as high concentration osmolytes in some certain deep-sea invertebrates and can also be used as carbon, nitrogen, or energy sources by bacteria [77, 78]. Taurine/hypotaurine transporter genes and degradation pathways for hypotaurine and taurine were found that release L-alanine (which may be converted to pyruvate or degraded to yield ammonium) and pyruvate that can be fed directly into central carbon metabolism. The sulfoacetaldehyde and sulfinoacetaldehyde produced as end products of taurine/hypotaurine degradation may also be further metabolized to acetyl-CoA and acetate with the concomitant production of sulfite. It has been shown that SAR11 can grow on taurine alone [79], and taurine has been suggested as a major source of C, N and S for SAR11 in the marine habitat [80]. The presence of these pathways suggests that at least some SAR202 members are also capable of utilizing taurine and hypotaurine as carbon sources.

One of the most readily available form of reduced sulfur in the euphotic zone of the open ocean is dimethylsulfoniopropionate (DMSP), an organosulfur compound produced by marine algae as compatible solute [81]. the main source of DMSP in the deeper parts of the water column is sedimentation of particulate DMSP and DMSP containing cells [81, 82]. While most DMSP is demethylated and utilized for amino acid biosynthesis (e.g., SAR11 and *Roseobacter* clade) [83], an alternative degradation pathway allows cleavage to 3-hydroxypropionate and dimethylsulfide (DMS) by the action of DMSP lyase (*dddD*) (e.g., SAR11) [84]. Several bacterial genera, e.g., *Rhodococcus, Acinetobacter, Pseudomonas* etc. are able to use DMS as sole sulfur source. As only one SAG encoded a DMSP lyase (Alt-SCGC-AB629-P13), and coupled with the uncertain knowledge on availability of DMSP in deeper waters, this process is unlikely to be widespread in the deep strata. However, several key downstream genes for oxidation of DMS itself, leading to the production of methanesulfonates, sulfite and formaldehyde were found (See Figs. 3 and 4). Apart from the biological production of methanesulfonates, chemical oxidation of atmospheric DMS can also lead to methanesulfonate production (several megatons), which is ultimately deposited in the oceans. Methanesulfonate transporters, methanesulfonate monoxygenases and formate dehydrogenases, which would allow the use of methanesulfonates as an energy source, were found (Figs. 3 and 4). However genes for formaldehyde assimilation (found in methylotrophs) were absent in SAR202 genomes.

While the deep marine habitat is considered to be largely oxic, local oxygen-deprived conditions favoring anaerobic respiration can exist in, e.g., particulate matter, oxygen minimum zones, and deep-sea vents and cold seeps characterized by the emission of reduced fluids. While most components of the aerobic respiratory chain were detected in SAR202 genomes (see Fig. 3), the widespread presence of tetrathionate reductases (at least 15 MAGs and 1 SAG) that can reduce tetrathionate to thiosulfate under anaerobic conditions, suggests the possibility for facultative anaerobic respiration [85]. Furthermore, thiosulfate/sulfate transporters were found, that may help import thiosulfate, opening a possibility for the enzyme rhodanese to metabolize thiosulfate [86, 87]. The enzyme rhodanese is widely distributed in prokaryotes [88] but its actual role is still debated, and suggestions span from involvement in cyanide detoxification to sulfur metabolism. However, its activity also results in the production of sulfite [86].

The degradation of taurine, hypotaurine, methanosulfonate, DMS and rhodanese activity all would release sulfite, which is highly reactive. Sulfite may be oxidized by two distinct dissimilatory oxidation pathways, either via the SOX system [89] or by *apr* (adenylylsulfate reductase) [78, 90]. No genes for the SOX system were found, but several genomes encoded the *apr* gene (Figs. 3 and 4) that converts sulfite to adenosine 5’-phosphosulfate (APS). It has been suggested that the phylogeny of the *aprA* gene can discriminate sulfur-oxidizing (SOB) and sulfate-reducing (SRB) bacteria [91, 92]. However, all SAR202 *aprA* genes form a separate clade, which also includes an *aprA* gene found in a euryarchaeal fosmid (KM3-67-G08), originating from metagenomic fosmid libraries of the Ionian Sea at 3000 m depth [93] (Supplementary Fig. S9). The closest relatives of this clade all originate from MAGs of sediment microorganisms (Chloroflexi bacterium CSP1-4 [94] and the *Candidatus* Rokubacteria, representative of a new phylum [94, 95]. In our case, given that the deep ocean is largely oxic, and that we found no evidence of dissimilatory sulfite reductases (*dsr*) genes in these genomes, the *aprA* genes from SAR202 likely represent a new group of sulfur-oxidizing *aprA* genes. While *aprA* genes have also been reported in deep specific SAR11 SAGs [15], they cluster together with the *aprA* genes of surface SAR11 genomes in the sulfur-oxidizing proteobacteria lineage I of *aprA* phylogeny (Supplementary Fig. S9). These genes were proposed to participate in taurine metabolism to detoxify the highly reactive sulfite (producing APS) and play a key role in the sulfur metabolism in deep specific SAR11 bacteria [15].

However, to produce ATP from sulfite via the activity of *aprBA* genes, a second step involving conversion of APS to sulfate is required. The gene encoding the corresponding enzyme (*sat*, sulfate adenylyltransferase) was also identified in SAR202 genomes. This would allow SAR202 to utilize the excess sulfite produced as a by-product of organosulfur compound metabolism as an energy source, producing sulfate as a final product. It is unclear if the complete sulfate assimilation pathways are present in SAR202, as only a single phosphoadenosine phosphosulfate (PAPS) reductase was found in one MAG (Casp-Chloro-G4) and no sulfite reductases were found. It may be that SAR202, similar to SAR11, rely on availability of reduced sulfur compounds [15, 96–98].

## Conclusions

The dark ocean is several times larger than the photic zone in sheer size and the majority of its microbial inhabitants are unknown. In this work, we have reconstructed genomes of the uncultivated SAR202 cluster (phylum *Chloroflexi*), which appears particularly abundant in the aphotic zone of global oceans. Moreover, the distribution of SAR202 sequences in the currently available deep metagenomic datasets (*n* = 50) suggests that SAR202 populations are vertically stratified, with distinct mesopelagic and bathypelagic groups. In particular, at least five genomes (belonging to only two distinct species) appear to be widely distributed in geographically distant regions in the bathypelagic zone of the world’s oceans.

Based on the higher abundance of the SAR202 cluster in the metagenomes of 0.–0.8 µm size fraction in the MALASPINA datasets, it is expected that these are free living microbes. Genome size estimations suggested sizes in the range of 2.5–3 Mb, that is in line with the large cell sizes observed by FISH [10]. Their larger cell-sizes coupled with higher abundances in the deep ocean suggest they alone make up for a significant fraction of the total biomass in this oligotrophic habitat. It must be considered however that total cell numbers are an order of magnitude lower in the deeper oceans than at the surface [99]. The genomes of the SAR202 group described here contain several pathways for metabolizing multiple organosulfur compounds as carbon, nitrogen and sulfur sources, which in turn implies that at least some of these compounds (e.g., DMS, DMSP, taurine, hypotaurine) may be more widely available in the deep ocean than suspected before [100, 101]. There are multiple sources of sulfur containing compounds, e.g., DMSP in sinking particulate cells (algal or microbial) from the upper layers, release of osmolytes from deep marine invertebrates (e.g., taurine, hypotaurine), production by heterotrophic microbes [103], hydrogen sulfide emissions from deep-sea vents, cold seeps and ocean sediments, especially at continental margins, or others that are as yet unknown. Similar disparities also exist in the measurements of total carbon input into this realm that do not match the higher estimated respiration rates [99], illustrating our as yet incomplete understanding of the remineralization processes and the participating entities at work in the dark ocean. In this work, we present initial evidence that hints at the presence of multiple, broadly available sulfur compounds in the deep sea that may be used by the abundant SAR202 group both as carbon and energy sources. Moreover, multiple degradation pathways found in these genomes appear to converge upon the production of sulfite, that may be oxidized to sulfate by adenylylsulfate reductase, suggesting that SAR202 are sulfite-oxidizers, making them key players in the sulfur cycle at the deep marine environment.

## Electronic supplementary material

supplementary figures

Supplementary table S1

Supplementary table S2

Supplementary Table S3

Supplementary table S4

Supplementary table S5
