# Supplementary material for: The enigmatic SAR202 cluster up close: shedding light on a globally distributed dark ocean lineage involved in sulfur cycling
Source: ISME J. 2017 Dec 5;12(3):655–68. doi: 10.1038/s41396-017-0009-5 (PMC5864207; doi:10.1038/s41396-017-0009-5)
Supplement: Supplementary file 1 — supplementary figures [file 41396_2017_9_MOESM1_ESM.docx]

**The enigmatic SAR202 cluster up close: shedding light on a globally distributed dark ocean lineage involved in sulfur cycling**

Maliheh Mehrshad^1^, Francisco Rodriguez-Valera^2^, Mohammed Ali Amoozegar^3^, Purificación López-García^4^, Rohit Ghai^1^*

^1^Institute of Hydrobiology, Department of Aquatic Microbial Ecology, Biology Centre of the Academy of Sciences of the Czech Republic, České Budějovice, Czech Republic

^2^Evolutionary Genomics Group, Universidad Miguel Hernández, San Juan de Alicante, Spain

^3^ Extremophiles Laboratory, Department of Microbiology, Faculty of Biology and Center of Excellence in Phylogeny of Living Organisms, College of Science, University of Tehran, Tehran, Iran

^4^ Ecologie, Systématique, Evolution, CNRS, Université Paris-Sud, Université Paris-Saclay, AgroParisTech, Orsay, France

*Corresponding author:

Rohit Ghai

Institute of Hydrobiology, Department of Aquatic Microbial Ecology, Biology Centre ASCR

Na Sadkach 7, 370 05, České Budějovice, Czech Republic

Tel: 00420 38777 5819

Email:ghai.rohit@gmail.com

Subject Category: Integrated genomics and post-genomics approaches in microbial ecology


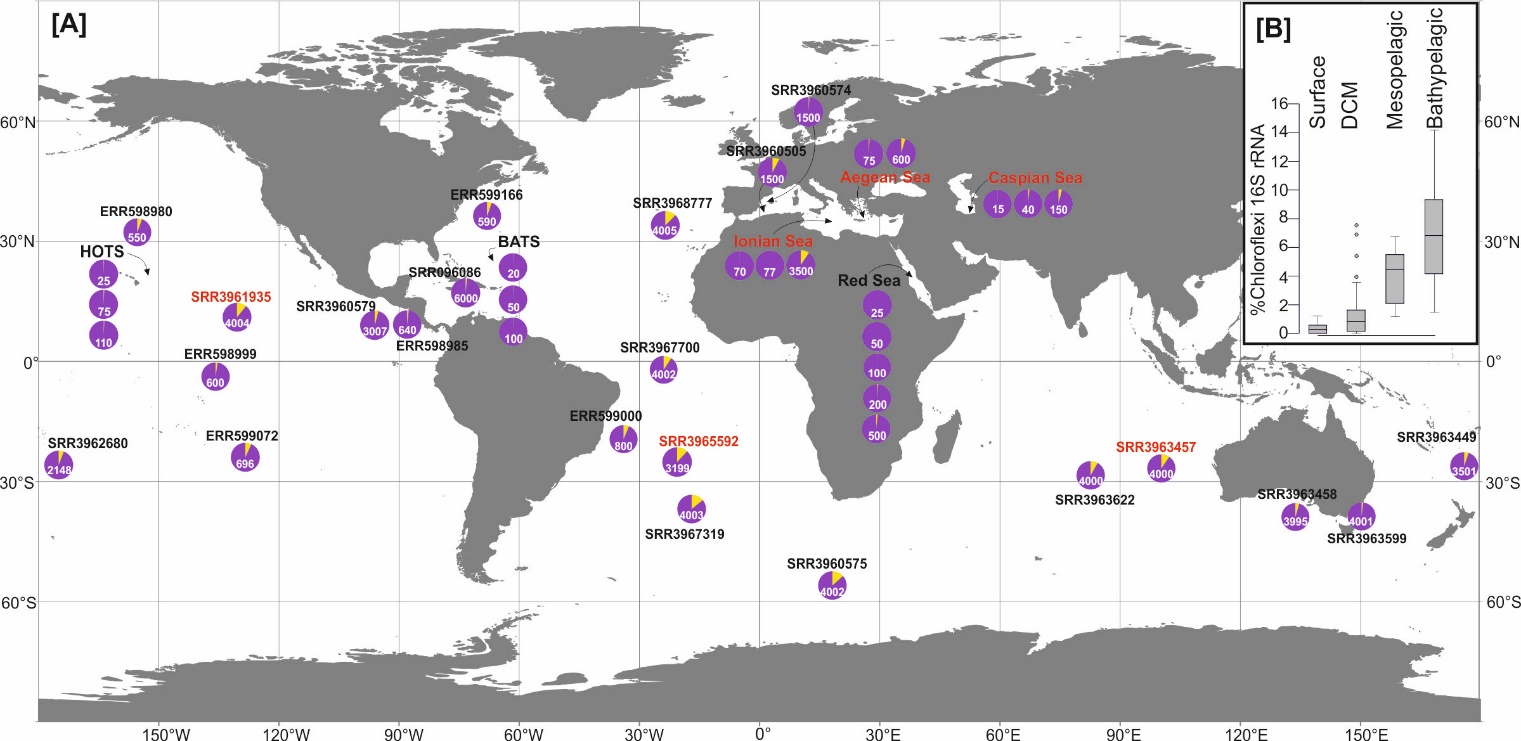


**Supplementary Figure S1-** **[A]** Percentage of *Chloroflexi* related 16S rRNA reads in marine and brackish prokaryotic communities based on unassembled metagenomic datasets. Brackish datasets include three different depths of the Caspian Sea. Marine datasets include Aegean Sea (one DCM and one deep dataset), Ionian Sea (one DCM and one deep dataset), Atlantic BATS, Pacific HOTS and Red Sea depth profile datasets together with selected deep datasets from MALASPINA and TARA expeditions and the Puerto Rico deep trench dataset. The sample depth for each dataset is mentioned inside the pie chart. The *Chloroflexi* fraction is shown in yellow within the pie charts that are placed at their approximate geographical position of their origin. Datasets highlighted in red were used for the assembly. **[B]** Box plot analysis of 16S rRNA reads assigned to *Chloroflexi* in 20 million read subsets of the metagenomes from different depths of marine environments including surface (TARA surface datasets (n=17) and the Caspian Sea 15m dataset) DCM (TARA DCM datasets (n=46) together with the eastern and western Mediterranean and also the Caspian Sea 40m datasets), mesopelagic (Datasets cover the depths from 150m to 1000 m (n=19) including Caspian Sea (150m), TARA mesopelagic (550m-1000m), Aegean Sea (600m) and Marmara Sea (1000m)), and Bathypelagic (depth range 1500m to 6000m (n=31) including deep MALASPINA datasets together with the Ionian Sea deep dataset (3500m) Puerto Rico deep trench dataset (6000m)). The black dots in the box plots are outlier values and the horizontal line within the indicates the median value.


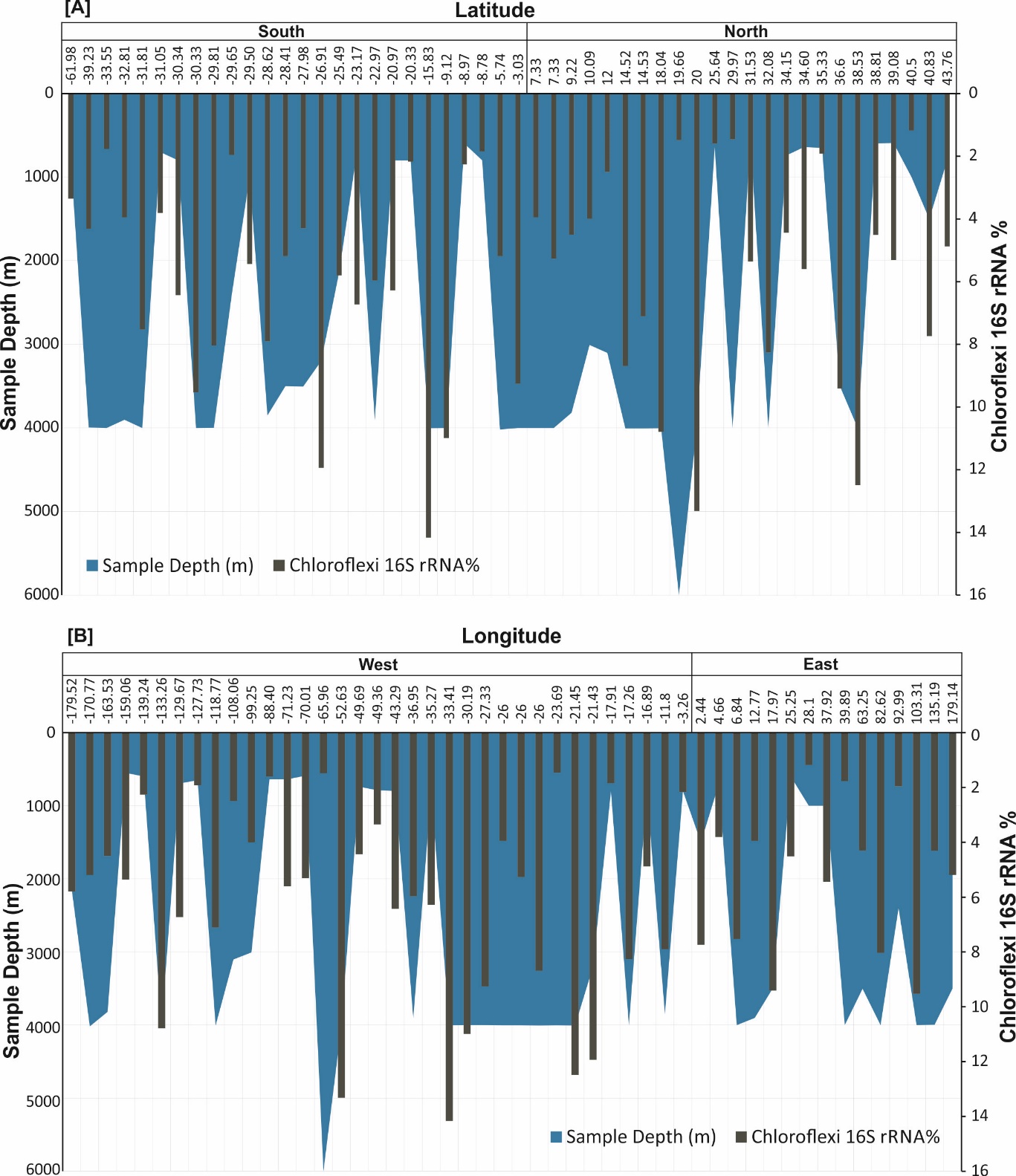


**Supplementary Figure S2-** Distribution of *Chloroflexi* 16S rRNA reads (as % of total microbial community) shown with respect to depth at different **[A]** latitude and **[B]** longitude of the sampling site. The complete list of datasets used is available in Supplementary Table S3.


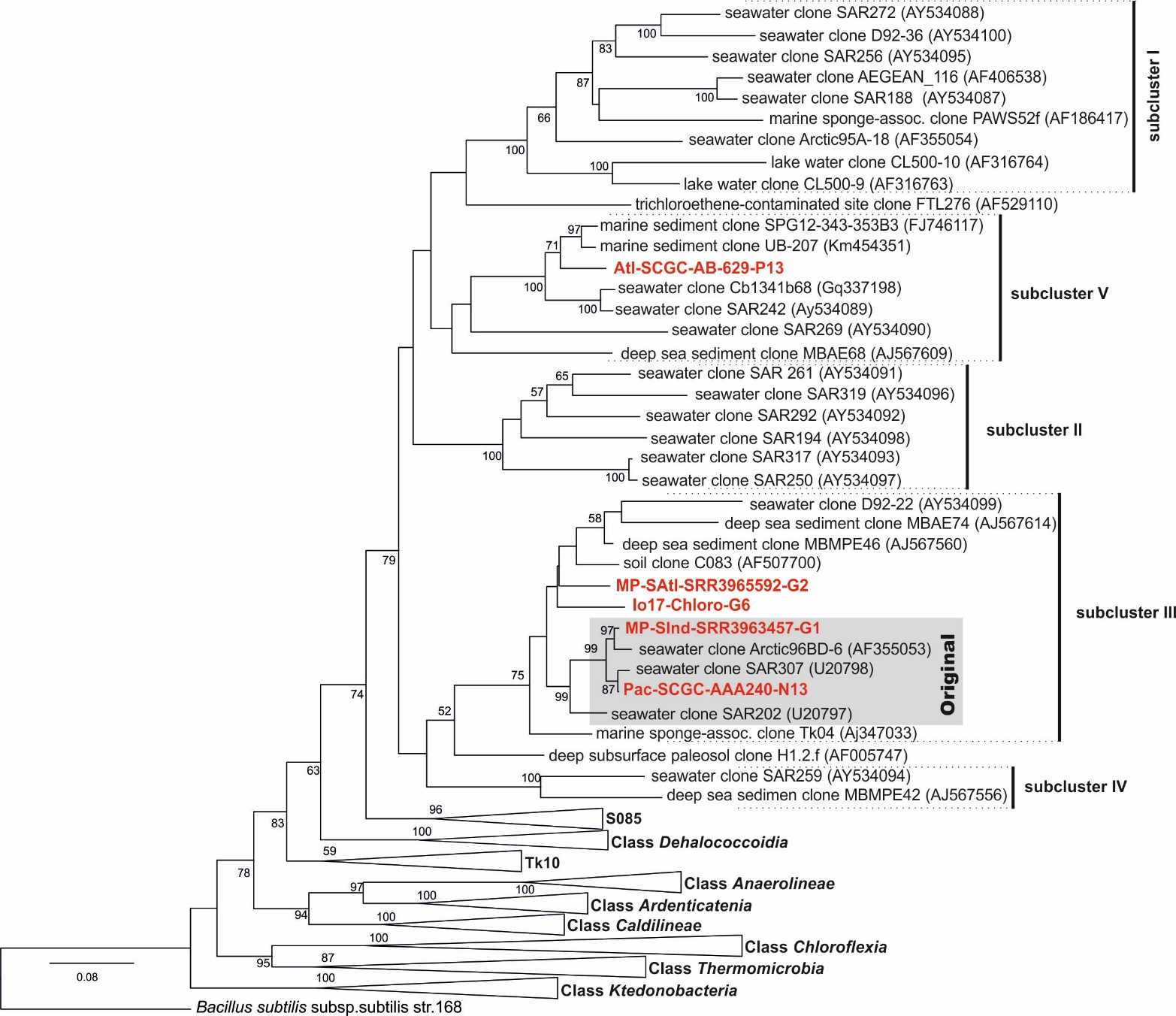


**Supplementary Figure S3-** Maximum likelihood phylogeny of 16S rRNA genes of SAR202 cluster together with the 16S rRNA genes of available single cell genomes of the cluster and 16S rRNA genes present in reconstructed MAGs of SAR202 cluster (shown in red) in comparision with other members of the phylum *Chloroflexi*. The original SAR202 related clones are shown inside the gray box. Bootstrap values (%) are indicated at the base of each node. 16S rRNA gene from *Bacillus subtilis* subsp.subtilis str.168 was used as outgroup.


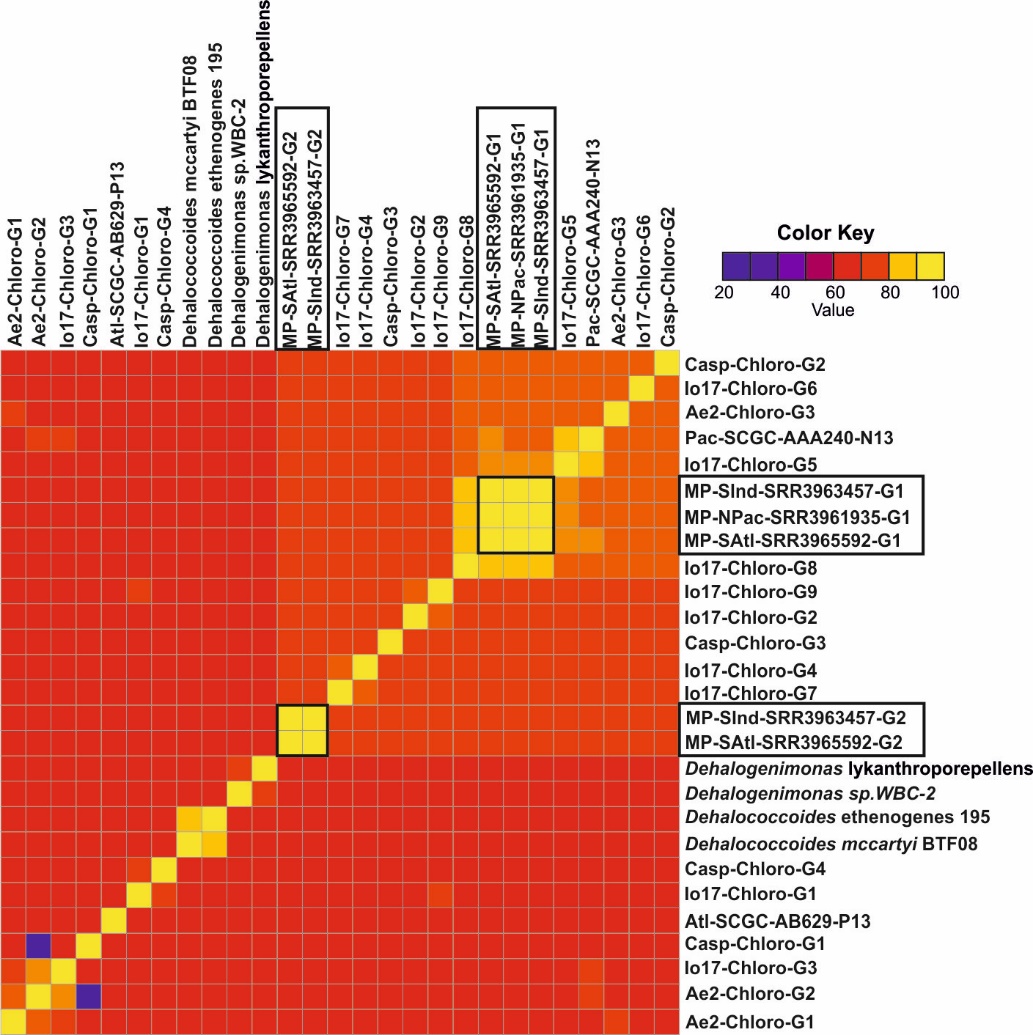


**Supplementary Figure S4-** Average nucleotide comparison (ANI) heat map of SAR202 MAGs and SAGs. Reconstructed genomes belonging to the same species are highlighted by a black boundary. A color key is shown at the top right.


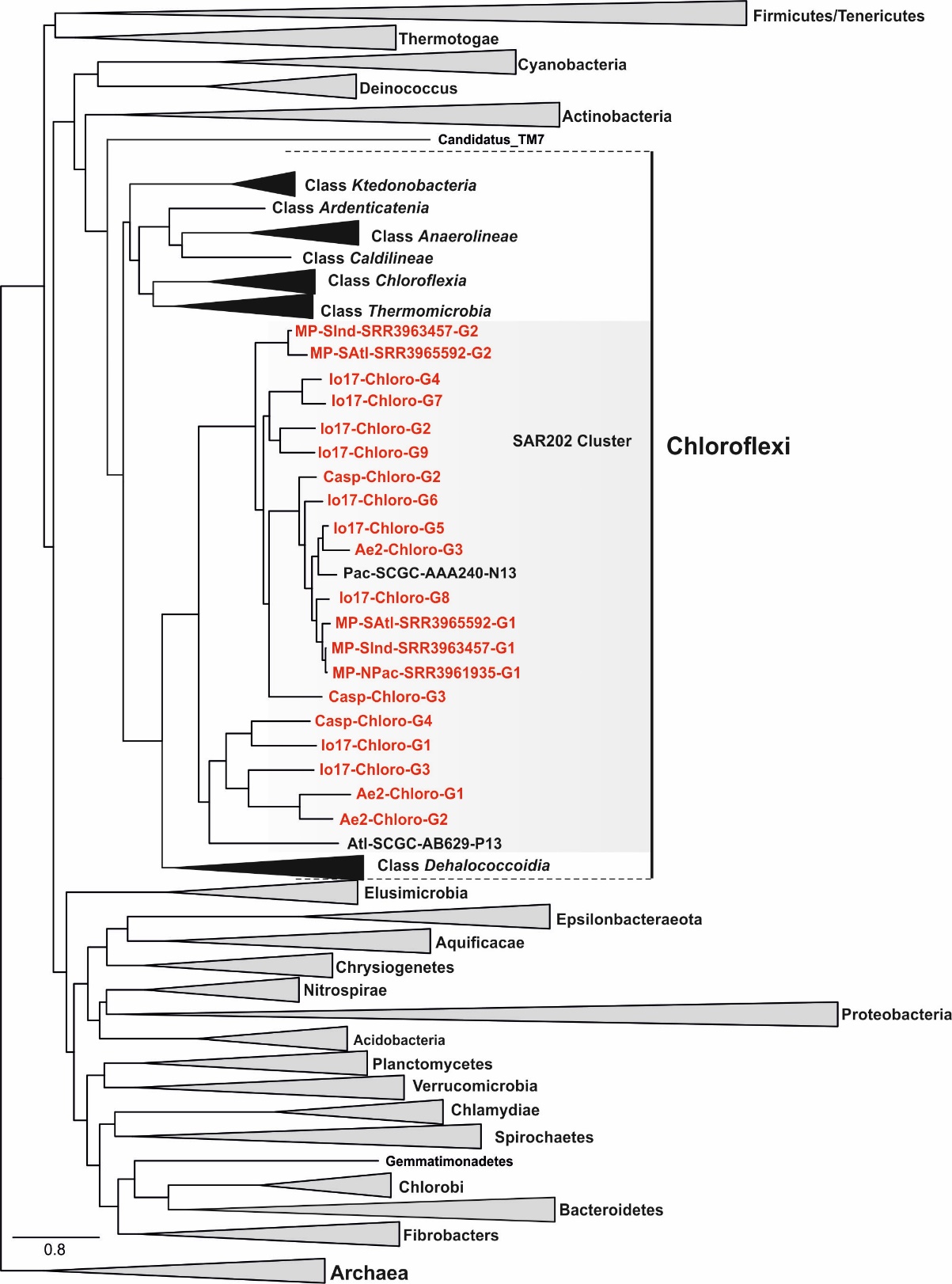


**Supplementary Figure S5-** Maximum likelihood reference phylogenomic tree constructed by adding the complete genomes of representatives from all known *Chloroflexi* classes, two SAGs and reconstructed MAGs of this study (except for the Casp-chloro-G1) to the built-in tree of life in PhyloPhlAN. Representatives of the SAR202 cluster are placed in a gray box. The metagenome assembled genomes (MAG) are shown in red.

**
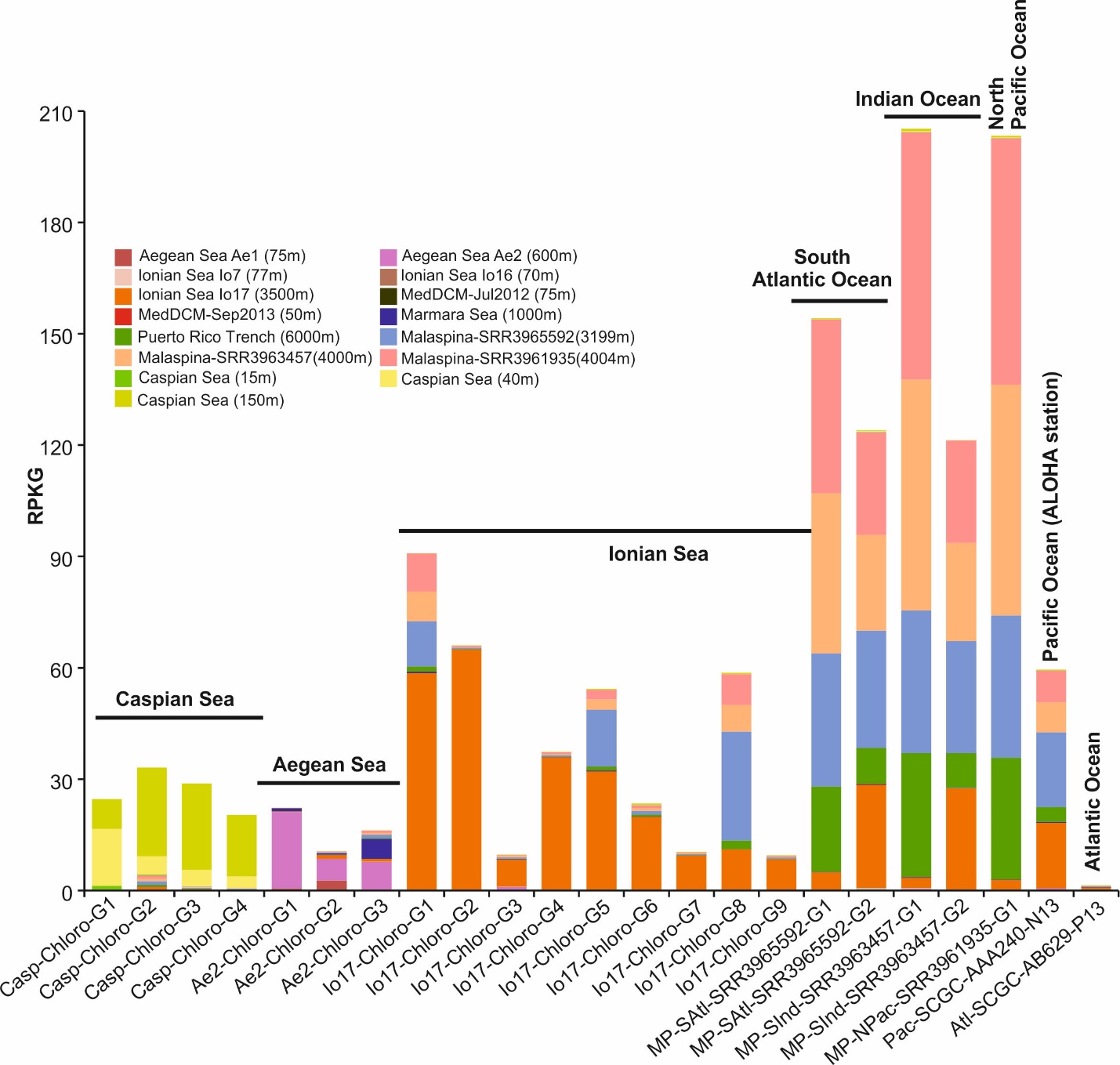
**

**Supplementary Figure S6-** Metagenomic recruitment of all reconstructed genomic bins in SAR202 cluster from different data sets of Caspian, Aegean and Ionian Seas and MALASPINA SRR3965592, SRR3963457, and SRR3961935 against different brackish and marine datasets. Brackish datasets include three different depths of the Caspian Sea. Marine datasets include Mediterranean DCM datasets of 2012 and 2013, Aegean Sea DCM and deep datasets, with two Ionian Sea DCM and one deep dataset, Marmara Sea deep dataset, and Puerto Rico Trench deep dataset, together with three of the deep MALASPINA datasets used for assembly The sample depth for each dataset is mentioned within parentheses.


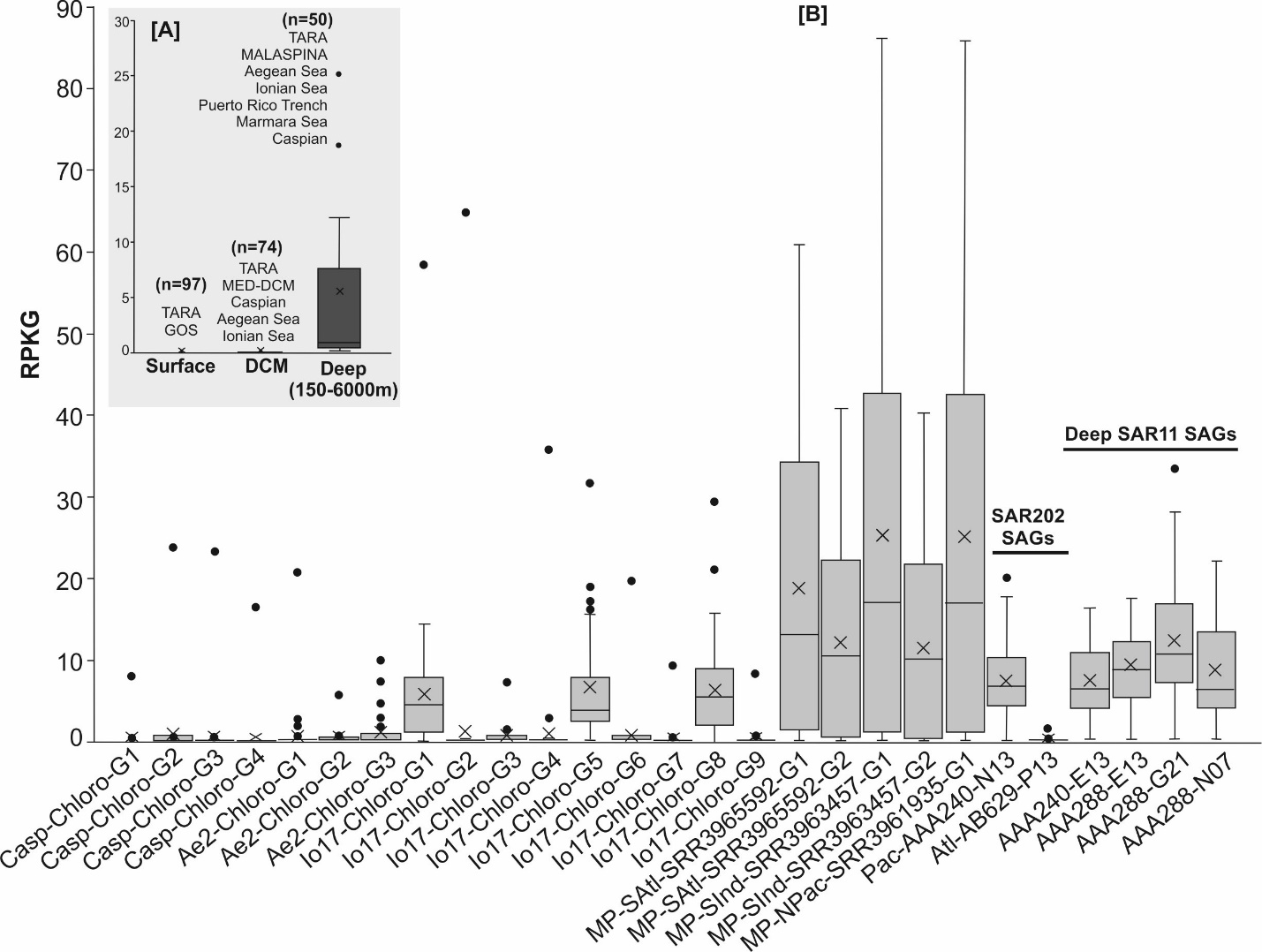


**Supplementary Figure S7-** Box plot overview of the recruitment (RPKG) of SAR202 cluster genomes. **[A]** comparison of the average RPKG values of all MAGs and SAGs of the SAR202 cluster in surface, DCM, and deep datasets. The datasets used for each depth and the total number of datasets are mentioned at the top of each box. **[B]** Overview of the recruitment RPKG distribution of all genomes in SAR202 cluster together with deep specific single cell amplified genomes of the SAR11 clade along deep datasets of TARA and MALASPINA expeditions, Caspian, Aegean, Ionian, and Marmara Sea and the Puerto Rico deep trench dataset (n=50). The black dots in the box plots are outlier values and the mean and median values of each distribution are represented by a cross and a horizontal line respectively.


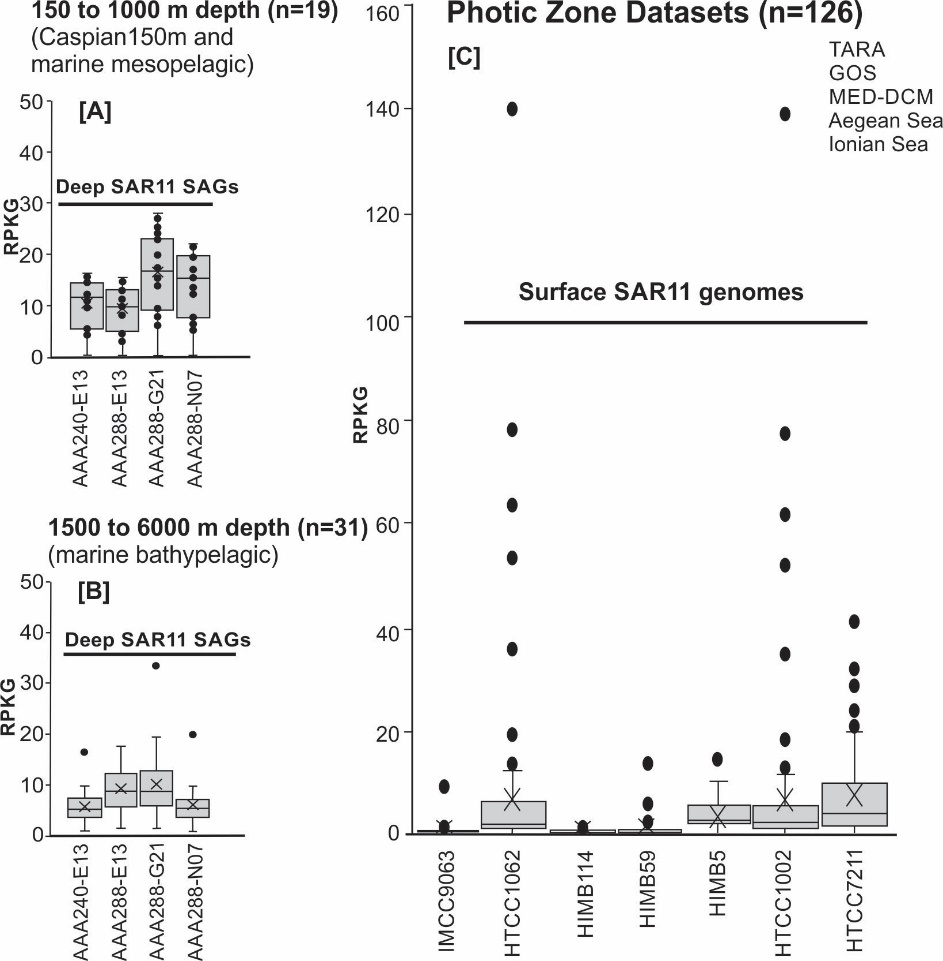


**Supplementary Figure S8-** Overview of the recruitment (RPKG) distribution of **[A]** deep specific single cell amplified genomes of the SAR11 clade in marine mesopelagic datasets together with the deep Caspian dataset from 150 m depth. Datasets cover the depths from 150m to 1000 m (n=19) in the mesopelagic layer and they include Caspian Sea (150m), TARA mesopelagic (550m-1000m), Aegean Sea (600m) and Marmara Sea (1000m) and **[B]** in marine deep datasets in the range of the 1500m to 6000m (n=31) including deep MALASPINA datasets together with the Ionian Sea deep dataset (3500m) Puerto Rico deep trench dataset (6000m). **[C]** Recruitment (RPKG) distribution of surface isolated genomes of the SAR11 clade in marine photic zone datasets from marine surface and DCM of TARA and GOS expeditions together with eastern and western Mediterranean DCM. The black dots in the box plots are outlier values.


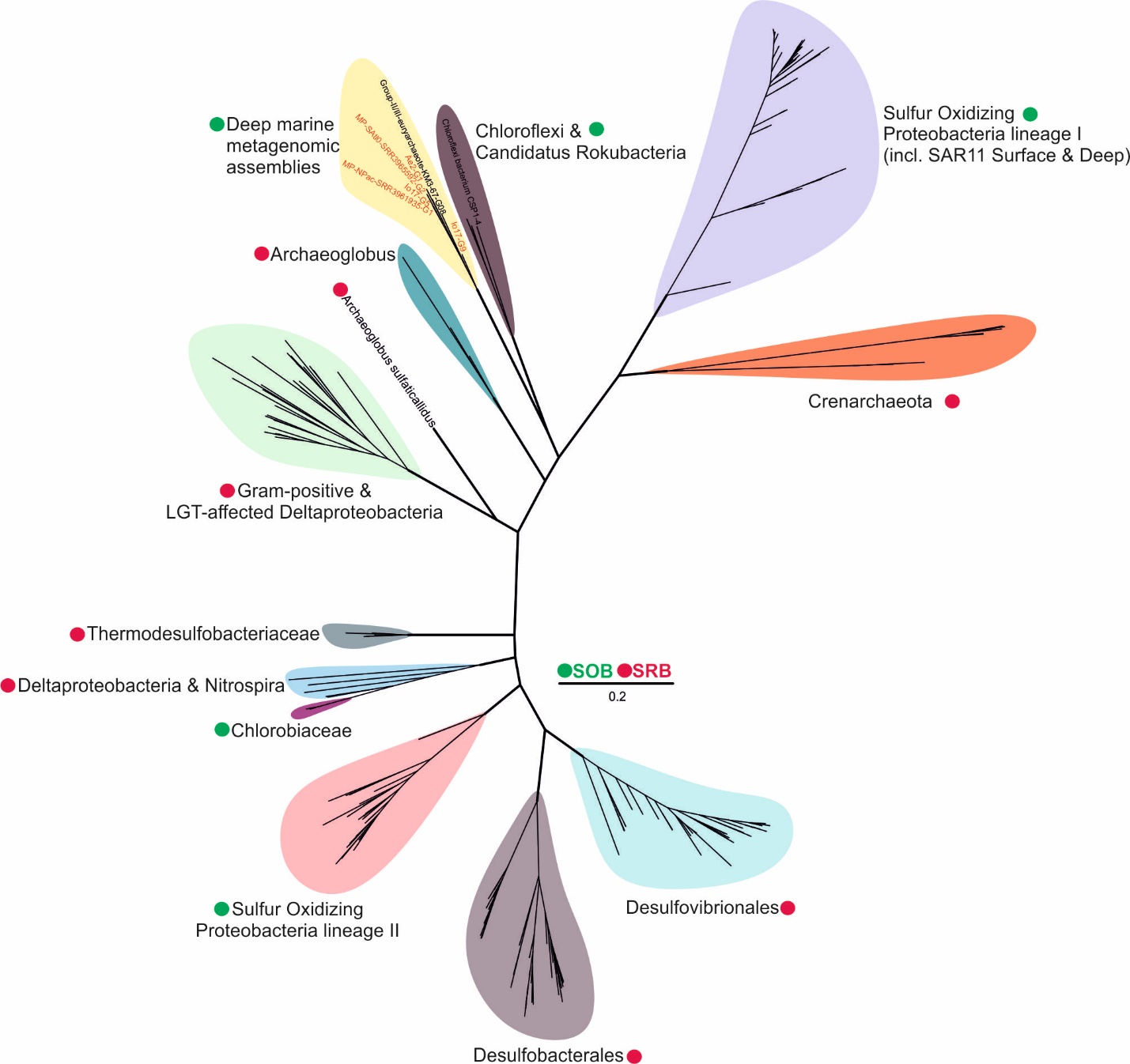


**Supplementary Figure S9-** Maximum likelihood tree of the *aprA* protein sequences from different bacteria and archaea. The *aprA* genes present in the members of the SAR202 cluster are highlighted in red. The reducing or oxidizing nature of the *aprA* genes in each clade is shown by red or green circles respectively. (SOB: Sulfur oxidizing bacteria/prokaryotes, SRB: Sulfur reducing bacteria/prokaryotes).
