# Supplementary material for: The enigmatic SAR202 cluster up close: shedding light on a globally distributed dark ocean lineage involved in sulfur cycling
Source: ISME J. 2017 Dec 5;12(3):655–68. doi: 10.1038/s41396-017-0009-5 (PMC5864207; doi:10.1038/s41396-017-0009-5)
Supplement: Supplementary file 2 — Supplementary table S1 [file 41396_2017_9_MOESM2_ESM.docx]

**Supplementary table S1:** Statistics of all datasets used for assembly from different environments

| Environment | Sample information | | | | Sequence information | | | | | | Community information |
| --- | --- | --- | --- | --- | --- | --- | --- | --- | --- | --- | --- |
|  | name | site | date | depth | Bioproject | Biosample | Run | #reads (Million) | Read length (bp) | #sequences (Gbp) | % Chloroflexi 16S rRNA |
| Caspian Sea | Caspian15m | 36.51 N 52.36 E | Oct2013 | 15 | PRJNA279271 | SAMN03444909 | SRR2026986 | 127.5 | 100 | 25.5 | 0.2 |
| Caspian Sea | Caspian40m | 36.51 N 52.36 E | Oct2013 | 40 | PRJNA279271 | SAMN03444948 | SRR2027816 | 159.9 | 100 | 32.0 | 1.66 |
| Caspian Sea | Caspian150m | 36.51 N 52.36 E | Oct2013 | 150 | PRJNA279271 | SAMN03444961 | SRR2027830 | 168.9 | 100 | 33.8 | 3.96 |
| Mediterranean Sea (DCM) | MedDCM-JUL2012 | 38.06 N 0.23 W | Jul2012 | 75 | PRJNA257723 | SAMN02954012 | SRR1539383 | 67.6 | 101 | 13.7 | 0.77 |
| Mediterranean Sea (DCM) | MedDCM-SEP2013 | 38.06 N 0.23 W | Sep2013 | 50 | PRJNA257723 | SAMN02954150 | SRR1539645 | 58.2 | 100 | 11.6 | 0.06 |
| Aegean Sea (DCM) | Ae1 | 38.81 N 25.25 E | Oct2010 | 75 | PRJNA305355 | SAMN04325109 | SRR2981519 | 58.3 | 100 | 11.7 | 1.09 |
| Aegean Sea | Ae2 | 38.81 N 25.25 E | Oct2010 | 600 | PRJNA305355 | SAMN04325110 | SRR2981523 | 130.2 | 100 | 26.0 | 4.51 |
| Ionian Sea (DCM) | Io7 | 36.48 N 15.65 E | Oct2010 | 77 | PRJNA305355 | SAMN04325106 | SRR2981506 | 111.1 | 100 | 22.2 | 0.08 |
| Ionian Sea (DCM) | Io16 | 36.59 N 18.69 E | Oct2010 | 70 | PRJNA305355 | SAMN04325107 | SRR2981512 | 77.5 | 100 | 15.5 | 0.15 |
| Ionian Sea | Io17 | 36.6 N 17.97 E | Oct2010 | 3500 | PRJNA305355 | SAMN04325108 | SRR2981515 | 97.6 | 100 | 19.5 | 9.41 |
| MALASPINA, South Atlantic Gyral Province | MP0556 | 26.91 S 21.43 W | Jan2011 | 3199 | PRJNA330027 | SAMN05422117 | SRR3965592 | 10.3 | 150 | 3.1 | 11.94 |
| MALASPINA, Indian South Subtropical Gyre Province | MP1202 | 30.33 S 103.31 E | March2011 | 4000 | PRJNA330001 | SAMN05422148 | SRR3963457 | 12.2 | 150 | 3.7 | 9.53 |
| MALASPINA, North Pacific Tropical Gyre Province | MP2016 | 18.04 N 133.26 W | May2011 | 4004 | PRJNA330009 | SAMN05422136 | SRR3961935 | 11.3 | 150 | 3.4 | 10.79 |
