# Supplementary material for: The enigmatic SAR202 cluster up close: shedding light on a globally distributed dark ocean lineage involved in sulfur cycling
Source: ISME J. 2017 Dec 5;12(3):655–68. doi: 10.1038/s41396-017-0009-5 (PMC5864207; doi:10.1038/s41396-017-0009-5)
Supplement: Supplementary file 4 — Supplementary Table S3 [file 41396_2017_9_MOESM4_ESM.docx]

| Bin Id | Marker lineage  # | genomes | markers | Marker sets | 0 | 1 | 2 | 3 | 4 | 5+ | Completeness |
| --- | --- | --- | --- | --- | --- | --- | --- | --- | --- | --- | --- |
| **Caspian Sea (15m, 40m, and 150m)** | | | | | | | | | | | |
| Casp-chloro-G1 | Chloroflexi (1) | 20 | 225 | 149 | 141 | 84 | 0 | 0 | 0 | 0 | 33.97 |
| Casp-chloro-G2 | Chloroflexi (1) | 20 | 225 | 149 | 53 | 171 | 1 | 0 | 0 | 0 | 74.54 |
| Casp-chloro-G3 | Chloroflexi (1) | 20 | 225 | 149 | 63 | 161 | 1 | 0 | 0 | 0 | 68.01 |
| Casp-chloro-G4 | Chloroflexi (1) | 20 | 225 | 149 | 71 | 153 | 1 | 0 | 0 | 0 | 58.5 |
| **Aegean Sea (Ae2, 600m)** | | | | | | | | | | | |
| Ae2-chloro-G1 | Chloroflexi (1) | 20 | 225 | 149 | 179 | 36 | 10 | 0 | 0 | 0 | 20.81 |
| Ae2-chloro-G2 | Chloroflexi (1) | 20 | 225 | 149 | 154 | 60 | 10 | 1 | 0 | 0 | 31.1 |
| Ae2-chloro-G3 | Chloroflexi (1) | 20 | 225 | 149 | 202 | 22 | 1 | 0 | 0 | 0 | 9.4 |
| **Ionian Sea (Io17, 3500m)** | | | | | | | | | | | |
| Io17-chloro-G1 | Chloroflexi (1) | 20 | 225 | 149 | 108 | 116 | 1 | 0 | 0 | 0 | 43.62 |
| Io17-chloro-G2 | Chloroflexi (1) | 20 | 225 | 149 | 89 | 136 | 0 | 0 | 0 | 0 | 55.48 |
| Io17-chloro-G3 | Chloroflexi (1) | 20 | 225 | 149 | 21 | 64 | 118 | 22 | 0 | 0 | 88.7 |
| Io17-chloro-G4 | Chloroflexi (1) | 20 | 225 | 149 | 55 | 166 | 4 | 0 | 0 | 0 | 68.23 |
| Io17-chloro-G5 | Chloroflexi (1) | 20 | 225 | 149 | 56 | 167 | 2 | 0 | 0 | 0 | 70.25 |
| Io17-chloro-G6 | Chloroflexi (1) | 20 | 225 | 149 | 43 | 177 | 5 | 0 | 0 | 0 | 80.29 |
| Io17-chloro-G7 | Chloroflexi (1) | 20 | 225 | 149 | 78 | 146 | 1 | 0 | 0 | 0 | 54.76 |
| Io17-chloro-G8 | Chloroflexi (1) | 20 | 225 | 149 | 109 | 114 | 2 | 0 | 0 | 0 | 47.49 |
| Io17-chloro-G9 | Chloroflexi (1) | 20 | 225 | 149 | 24 | 70 | 78 | 44 | 7 | 2 | 86.69 |
| **MALASPINA, South Atlantic Gyral Province (SRR3965592, 3199m)** | | | | | | | | | | | |
| MP-SAtl-SRR3965592-G1 | Chloroflexi (1) | 20 | 225 | 149 | 83 | 90 | 52 | 0 | 0 | 0 | 58.1 |
| MP-SAtl-SRR3965592-G2 | Chloroflexi (1) | 20 | 225 | 149 | 163 | 62 | 0 | 0 | 0 | 0 | 29.62 |
| **MALASPINA, Indian South Subtropical Gyre Province (SRR3963457, 4000m)** | | | | | | | | | | | |
| MP-SInd-SRR3963457-G1 | Chloroflexi (1) | 20 | 225 | 149 | 51 | 172 | 2 | 0 | 0 | 0 | 70.92 |
| MP-SInd-SRR3963457-G2 | Chloroflexi (1) | 20 | 225 | 149 | 108 | 117 | 0 | 0 | 0 | 0 | 43.51 |
| **MALASPINA, North Pacific Tropical Gyre Province (SRR3961935, 4004m)** | | | | | | | | | | | |
| MP-NPac-SRR3961935-G1 | Chloroflexi (1) | 20 | 225 | 149 | 68 | 155 | 2 | 0 | 0 | 0 | 65.55 |

Supplementary Table S3: CheckM analysis of the SAR202 reconstructed MAGs
