# Supplementary material for: The enigmatic SAR202 cluster up close: shedding light on a globally distributed dark ocean lineage involved in sulfur cycling
Source: ISME J. 2017 Dec 5;12(3):655–68. doi: 10.1038/s41396-017-0009-5 (PMC5864207; doi:10.1038/s41396-017-0009-5)
Supplement: Supplementary file 6 — Supplementary table S5 [file 41396_2017_9_MOESM6_ESM.docx]

**Supplementary table S5:** Statistics of *Chloroflexi* genomic fragments in assemblies of different environments

|  | **% 16S rRNA**  **in raw reads** | **#Contigs** | **Largest**  **contig size**  **(kb)** | **Average**  **contig size**  **(kb)** | **Total size(Mb)** | **# reconstructed genomic bins**  **(#almost complete)** |
| --- | --- | --- | --- | --- | --- | --- |
| **Caspian Sea** | 0.2 (caspian 15m)  1.66 (Caspian 40m)  3.96 (Caspian 150m) | 205 | 96 | 25 | 5.1 | 4(3) |
| **MedDCM-Jul2012** | 0.77 | 33 | 226 | 16 | 0.53 | - |
| **MedDCM-Sep2013** | 0.06 | 4 | 8.9 | 6 | 0.02 | - |
| **Aegean Sea Ae1** | 1.09 | 3 | 7.3 | 6.6 | 0.02 | - |
| **Aegean Sea Ae2** | 4.51 | 111 | 36 | 14.7 | 1.6 | 3 |
| **Ionian Sea Io7** | 0.08 | 20 | 19 | 7.7 | 0.15 | - |
| **Ionian Sea Io16** | 0.15 | 3 | 7.9 | 7 | 0.02 | - |
| **Ionian Sea Io17** | 9.41 | 709 | 276 | 25 | 17.8 | 9(8) |
| **MALASPINA SRR3965592**  **South Atlantic Ocean** | 11.94 | 161 | 45 | 15.8 | 2.5 | 2(1) |
| **MALASPINA SRR3963457**  **South Indian Ocean** | 9.53 | 114 | 120 | 25.5 | 2.9 | 2(1) |
| **MALASPINA SRR3961935**  **North Pacific Ocean** | 10.79 | 66 | 143 | 34.5 | 2.3 | 1(1) |

*Gray rows are showing datasets with no Chloroflexi bin
